# Immune Escape Variants of H9N2 Influenza Viruses Containing Deletions at the Hemagglutinin Receptor Binding Site Retain Fitness *In Vivo* and Display Enhanced Zoonotic Characteristics

**DOI:** 10.1128/JVI.00218-17

**Published:** 2017-06-26

**Authors:** Thomas P. Peacock, Donald J. Benton, Joe James, Jean-Remy Sadeyen, Pengxiang Chang, Joshua E. Sealy, Juliet E. Bryant, Stephen R. Martin, Holly Shelton, Wendy S. Barclay, Munir Iqbal

**Affiliations:** a The Pirbright Institute, Pirbright, Woking, United Kingdom; b Department of Virology, Imperial College London, London, United Kingdom; c The Francis Crick Institute, London, United Kingdom; d Royal Veterinary College, University of London, London, United Kingdom; e Oxford University Clinical Research Unit and Wellcome Trust Major Overseas Programme, National Hospital of Tropical Diseases, Hanoi, Vietnam; f Structural Biology Science Technology Platform, The Francis Crick Institute, London, United Kingdom; Icahn School of Medicine at Mount Sinai

**Keywords:** avian influenza virus, H9N2, hemagglutinin, receptor binding site, zoonotic, pandemic, antigenic drift

## Abstract

H9N2 avian influenza viruses are enzootic in poultry across Asia and North Africa, where they pose a threat to human health as both zoonotic agents and potential pandemic candidates. Poultry vaccination against H9N2 viruses has been employed in many regions; however, vaccine effectiveness is frequently compromised due to antigenic drift arising from amino acid substitutions in the major influenza virus antigen hemagglutinin (HA). Using selection with HA-specific monoclonal antibodies, we previously identified H9N2 antibody escape mutants that contained deletions of amino acids in the 220 loop of the HA receptor binding sites (RBSs). Here we analyzed the impact of these deletions on virus zoonotic infection characteristics and fitness. We demonstrated that mutant viruses with RBS deletions are able to escape polyclonal antiserum binding and are able to infect and be transmitted between chickens. We showed that the deletion mutants have increased binding to human-like receptors and greater replication in primary human airway cells; however, the mutant HAs also displayed reduced pH and thermal stability. In summary, we infer that variant influenza viruses with deletions in the 220 loop could arise in the field due to immune selection pressure; however, due to reduced HA stability, we conclude that these viruses are unlikely to be transmitted from human to human by the airborne route, a prerequisite for pandemic emergence. Our findings underscore the complex interplay between antigenic drift and viral fitness for avian influenza viruses as well as the challenges of predicting which viral variants may pose the greatest threats for zoonotic and pandemic emergence.

**IMPORTANCE** Avian influenza viruses, such as H9N2, cause disease in poultry as well as occasionally infecting humans and are therefore considered viruses with pandemic potential. Many countries have introduced vaccination of poultry to try to control the disease burden; however, influenza viruses are able to rapidly evolve to escape immune pressure in a process known as “antigenic drift.” Previously, we experimentally generated antigenic-drift variants in the laboratory, and here, we test our “drifted” viruses to assess their zoonotic infection characteristics and transmissibility in chickens. We found that the drifted viruses were able to infect and be transmitted between chickens and showed increased binding to human-like receptors. However, the drift mutant viruses displayed reduced stability, and we predict that they are unlikely to be transmitted from human to human and cause an influenza pandemic. These results demonstrate the complex relationship between antigenic drift and the potential of avian influenza viruses to infect humans.

## INTRODUCTION

Avian influenza viruses (AIVs) pose a major threat to global food security, with emergent outbreaks causing billions of dollars of damage to affected countries (1). In addition to their economic impact on the poultry sector, AIVs also continue to pose a considerable threat to public health due to the emergence of zoonotic viruses with pandemic potential.

Low-pathogenicity avian influenza virus subtype H9N2 is one of the most widespread AIV subtypes in poultry. Despite the classification of H9N2 viruses as being of low pathogenicity, these viruses cause significant production losses in poultry in many countries throughout Asia, the Middle East, and North Africa (2, 3). In laboratory settings, H9N2 viruses typically exhibit low morbidity and mortality rates in chickens (4); however, H9N2 viruses have displayed flock mortality rates of up to 60% as well as moderately pathogenic phenotypes in laboratory settings (4–9). In recent years, an increasingly large number of human H9N2 infections have been identified in Hong Kong, Mainland China, Bangladesh, and Egypt, usually during surveillance for other zoonotic influenza virus infections such as H5N1, H5Nx, H7N9, and H10N8 (10–13). A growing body of serological evidence suggests that asymptomatic or mild infections with H9N2 may be fairly common in enzootic areas, especially among poultry workers (14). Experimental infections with H9N2 viruses in ferrets have demonstrated that some strains can exhibit transmission via respiratory droplets, a property generally thought to be a prerequisite to becoming a human pandemic influenza virus (15–20).

Classical inactivated vaccines have been widely utilized in poultry in many areas to attempt to curb economic losses associated with H9N2 infection (8, 21–24). Veterinary influenza vaccines face challenges similar to those of human seasonal influenza vaccines in terms of requiring continual evaluation and updating due to virus antigenic drift. To date, there are limited field data assessing the efficacy of currently licensed H9N2 vaccines, and epidemiological studies suggest that enzootic transmission may continue even in regions with large numbers of vaccinated flocks (21, 22, 24).

Hemagglutinin (HA) is a homotrimeric transmembrane glycoprotein encoded by segment 4 of the influenza virus genome and plays a major role in virus adaption to new hosts, antigenicity, and pathogenesis. Each monomer of HA consists of two domains: (i) a head that contains the receptor binding site (RBS), vital for the binding of influenza viruses to their cognate receptors of terminal sialic acid (SA) residues on glycans of the host cell surface, and (ii) the stalk domain, which contains the fusion peptide involved in the pH-mediated fusion of the viral and host endosomal membranes, allowing the release of virus genetic material into the host cytoplasm. Both natural immunity and vaccine-induced immunity are mediated primarily through antibodies against HA, in particular neutralizing antibodies that block the receptor binding ability of HA (25). In response to immune pressure exerted by neutralizing antibodies, viruses are able to “escape” through the accumulation of amino acid substitutions or, more rarely observed, amino acid deletions (26, 27).

The HA RBS is comprised of three subdomains, the 130 loop, the 190 helix, and the 220 loop. Residues within the 220 loop and, to a lesser extent, the 190 helix have been shown to be major determinants of the binding preference for either “human-like” α2,6-linked SA or “avian-like” α2,3-linked SA. Furthermore, these determinants of binding preference appear to be conserved across multiple influenza virus subtypes (28–31). Substitutions in amino acid residues that influence the preference for SA are well established as markers of augmented zoonotic and pandemic potential (32, 33).

In our previous study, we used a panel of monoclonal antibodies (MAbs) to select for H9N2 escape mutants of A/chicken/Pakistan/UDL-01/2008 (here referred to as UDL1/08). We demonstrated that a subset of these MAbs selected for mutant HA proteins that had deletions in the 220 loop regions of their RBSs, here collectively referred to as deletion mutants (34). Here we characterized the deletion mutants using a combination of *in vitro*, *in vivo*, and biochemical approaches to measure receptor binding avidity, replication kinetics, transmission potential in chickens, and HA stability. Our results help inform risk assessments of whether these viruses pose epizootic and zoonotic threats compared to contemporary H9N2 viruses found in poultry.

## RESULTS

### Deletion mutant viruses are poorly recognized by chicken and ferret antisera against homologous and heterologous H9N2 viruses.

**FIG 1:**
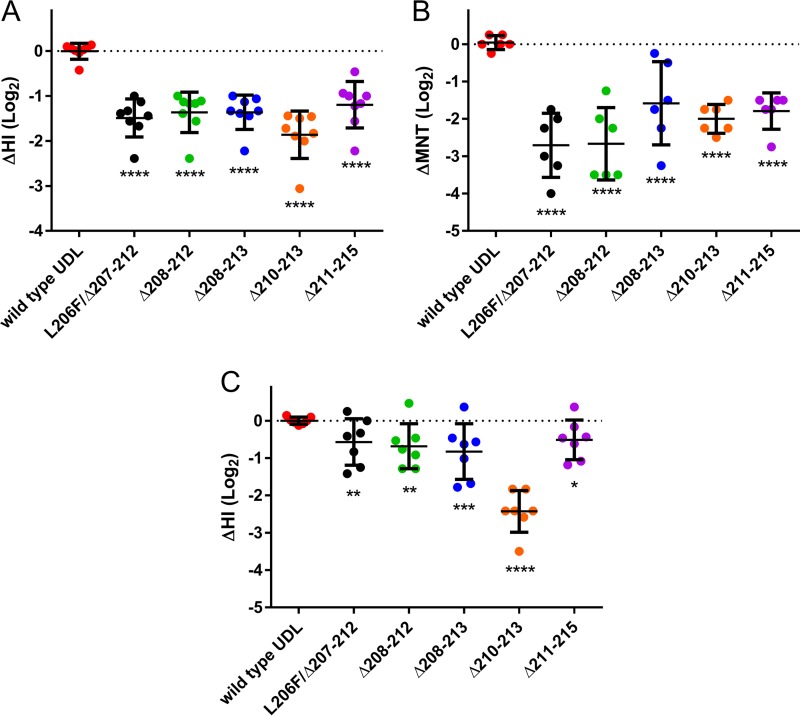
Antigenic properties of five 220 loop deletion viruses. (A) HI assay with 8 individual chicken polyclonal postinfection antisera raised against wt UDL1/08. The *y* axis indicates the relative HI titer compared to the homologous titer of the antisera against wt UDL1/08, normalized to zero (dotted line). (B) MNTs with 6 individual chicken polyclonal postinfection antisera raised against wt UDL1/08. The *y* axis indicates the relative MNT titer compared to the homologous titer of the antisera against wt UDL1/08, normalized to zero (dotted line). (C) 220 loop deletion viruses tested by relative HI titers with 6 heterologous antisera raised against Em/R66. Antisera against UDL1/08 were normalized to zero (dotted line). Each colored dot represents a different antiserum. Black lines show mean changes in HI and standard error bars for each mutant virus. Significance was determined by one-way ANOVA with multiple comparisons against wt UDL1/08. *, 0.05 ≥ *P* > 0.01; **, 0.01 ≥ *P* > 0.001; ***, 0.001 ≥ *P* > 0.0001; ****, *P* ≤ 0.0001.

We previously determined that all UDL1/08 viruses with loop deletions have greatly reduced hemagglutination inhibition (HI) activity against the selecting MAbs by >128-fold (34). To further evaluate the antigenic properties of these deletion mutants, we carried out HI and microneutralization (MNT) assays with a panel of 8 polyclonal chicken antisera raised against the wild-type (wt) UDL1/08 virus. All five deletion mutants exhibited a significant drop in their recognition by chicken polyclonal antisera, measured by both HI and MNT assays, compared to the wt virus (*P* ≤ 0.0001 by one-way analysis of variance [ANOVA] with multiple comparisons against the wt) (Fig. 1A and B and Table 1). The fold reductions in HI and MNT titers for the deletion mutant viruses compared to the wt UDL1/08 virus were between 2.3- and 3.6-fold and between 3.1- and 6.72-fold, respectively.

**TABLE 1:** Antigenic properties of five 220 loop deletion viruses^*a*^

Postinfection antiserum	Method	Geometric mean titer of virus (HA/MNT units)					
		Wild-type UDL1/08	L206F/Δ207–212	Δ208–212	Δ208–213	Δ210–213	Δ211–215
8× UDL1/08 (chicken p.i.)	HI	1,500	512****	583****	583****	412****	535****
	MNT	570	76.1****	114****	170****	128****	148****
6× Em/R66 (chicken p.i.)	HI	41.3	27.8****	22.6****	21.3****	10.9****	27.6****
1× BD/0994 (ferret p.i.)	HI	1,280^*b*^	320	2,560	320	320	2,560

a p.i., postinfection. **** indicates a *P* value of ≤0.0001, calculated by using one-way ANOVA with multiple comparisons against the wild-type titer.

b Significance was unable to be determined due to a single antiserum only.

In a field setting, vaccine seed strains are rarely closely antigenically matched to circulating poultry viruses, thus posing a continuing challenge for evaluating the efficacy of vaccine interventions (22, 35). Therefore, to assess whether vaccines would be able to afford protection if deletion mutant viruses arose, we performed HI assays using a panel of 6 separate chicken antisera raised against an antigenically distinct (34), older H9N2 isolate, A/chicken/Emirates/R66/2002 (here referred to as Em/R66). It was found again that all deletion mutants cross-reacted significantly less well than the wt UDL1/08 virus (*P* ≤ 0.0001) to the Em/R66 antisera (Fig. 1C and Table 1), with decreases of titers of between 1.4- and 5.4-fold. These results indicate that deletions within the 220 loop allow viruses to efficiently escape neutralizing antibodies such as those that may be generated by poultry vaccines.

Finally, we tested the antigenic cross-reactivity of the deletion mutant viruses with a single ferret antiserum raised against the H9N2 World Health Organization (WHO) candidate vaccine virus (CVV) seed strain most closely related to the wt UDL1/08 virus, A/Bangladesh/0994/2013 (BD/994) (36). We found that the L206F/Δ207–212 (a virus carrying the substitution L206F and a deletion of amino acids 207 to 212), Δ207–213, and Δ210–213 deletion mutants all had a 4-fold decrease in cross-reactivity compared to the wt UDL1/08 virus (Table 1). Surprisingly, the Δ208–212 and Δ211–215 deletion mutant viruses were inhibited 2-fold more efficiently by ferret BD/994 antisera than was the wt UDL1/08 virus.

### Deletion mutants retain replicative fitness in chicken cells *in vitro* and *ex vivo*.

**FIG 2:**
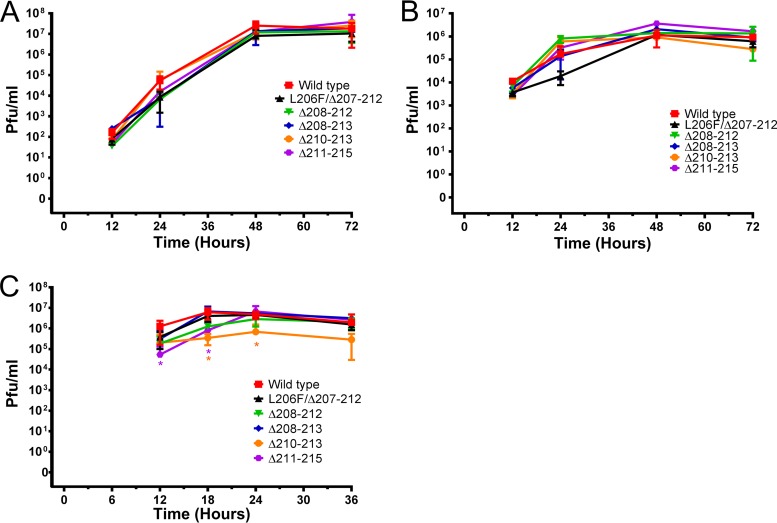
Replication dynamics of 220 loop deletion viruses *in vitro* and *ex vivo* in mammalian and avian cells and tissues. (A) MDCK cells infected at an MOI of 0.001. (B) Primary chicken kidney cells infected at an MOI of 0.01. (C) eTOCs infected with 240 PFU/eTOC.

To assess whether the deletion mutant viruses were attenuated *in vitro* and *ex vivo*, we performed several low-multiplicity-of-infection (MOI) growth curves in Madin-Darby canine kidney (MDCK) cells, chicken kidney cells (CKCs), and chicken embryonic tracheal organ cultures (eTOCs). The deletion mutants generally replicated to titers comparable to that of the wt virus in all cells and tissues tested (Fig. 2A to C). Two mutants, Δ211–215 and Δ210–213, however, showed some replicative attenuation across several time points in eTOCs.

Some variation in plaque sizes in MDCK cells between the different viruses was observed: the wt, Δ208–212, and Δ208–213 viruses displayed similar, large-plaque phenotypes, while the L206F/Δ207–212 and Δ211–215 viruses displayed slightly smaller plaques, and the Δ210–213 virus displayed a small-plaque phenotype (data not shown). However, virus plaque size did not correlate with replication in MDCK cells.

### Deletions in the 220 loop are stable after multiple egg passages and are viable in multiple H9N2 genetic backgrounds.

Previous studies investigating structural changes in HA, often after substitutions or deletions such as those described in this study, identified compensatory changes in either HA or neuraminidase (NA) that are selected in order to maintain viral fitness (37, 38). To investigate whether the deletion mutants would induce a similar pattern of HA or NA compensatory mutations, we performed 5 passages of our wt and deletion mutant viruses in embryonated chicken eggs. After passages 2 and 5, we sequenced the entire HA and NA coding regions and found that no further nonsynonymous substitutions occurred in either gene, with the sole exception of wt UDL1/08 virus HA1, which gained an N148K mutation, detectable by as early as passage 2. The role of this mutation has yet to be determined; however, we speculate that it may constitute egg adaptation.

Subsequently, we assessed whether the ability to tolerate deletions in the RBS was a universal or strain-specific trait of H9N2 viruses by evaluating the impact of loop deletions in both the BJ94 and American H9N2 lineages. Using reverse genetics, we generated a BJ94-lineage H9N2 virus, A/chicken/Wenzhou/606/2013, with both attempted deletions (Δ208–213 and Δ211–215) as well as an American-lineage H9N2 virus, A/turkey/Wisconsin/1/1966, with a Δ211–215 mutation. The successful rescue and replication of these additional mutants suggest that the ability to tolerate deletions in the RBS may indeed be a common trait of H9N2 HAs.

### A deletion mutant retains infectivity, pathogenicity, and transmissibility *in vivo* in chickens.

**FIG 3:**
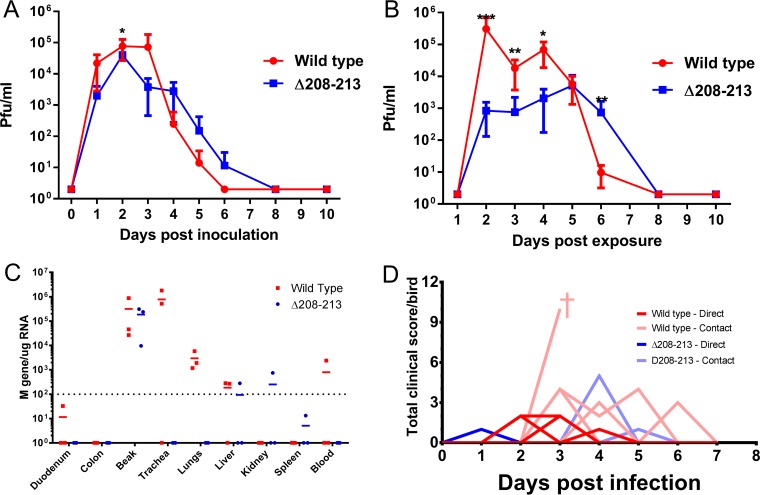
Comparative infectivity, shedding dynamics, transmission, tropism, and clinical presentation of the wt UDL1/08 and Δ208–213 viruses. (A) Mean oropharyngeal shedding of infectious virus by directly inoculated birds. For days 0 to 2, there were 7 birds per group; for day 3 onwards, there were 3 birds infected with the wt and 4 birds infected with deletion mutant due to culling and euthanization. Data show means ± standard errors. Student's *t* test was performed for comparisons between the viruses at each time point. *, 0.05 ≥ *P* > 0.01; **, 0.01 ≥ *P* > 0.001; ***, *P* ≥ 0.001. (B) Mean oropharyngeal shedding of infectious virus by contact birds (7 birds in each group throughout). (C) Presence of viral RNA in different tissues taken from directly infected birds at 2 days postinoculation. (D) Total clinical scores from individual birds recorded during the course of the experiment. † indicates culling of a bird for welfare reasons.

Little to no change in the replication of the deletion mutant viruses was observed *in vitro* and *ex vivo*. Therefore, a single mutant (Δ208–213) was selected to determine comparative infectivity, transmissibility, and pathogenicity in chickens. The Δ208–213 mutant was chosen from among the deletion mutants because it appeared to have the highest level of replication in eTOCs. Two groups of seven naive, specific-pathogen-free (SPF) chickens were inoculated intranasally with 10^6^ PFU of either the wt (UDL1/08) or the deletion mutant (Δ208–213) virus. At 24 h postinoculation, seven more naive birds were introduced (cohoused) with each group as contacts. Oropharyngeal and cloacal swabs were collected daily from each bird. Live-virus titration from oropharyngeal swabs indicated that both the wt and mutant viruses were comparatively infectious, since all birds exhibited robust shedding within 24 h postinoculation (Fig. 3A). In addition, all contact birds in both groups became infected at 1 day postcontact and shed infectious virus from the buccal cavity (Fig. 3B). Contact birds infected with the wt virus (UDL1/08) shed significantly larger amounts of virus from the buccal cavity on days 2 to 4, while the mutant virus (Δ208–213)-exposed contact birds shed significantly larger amounts of virus on day 5 postcontact. Directly inoculated birds in both groups, however, shed comparable levels of infectious virus from the buccal cavity throughout the experimental period (Fig. 3A). Although not statistically significant, 5/7 birds infected with the wt virus and 2/7 of their contacts shed infectious virus via the cloaca on one or more days, whereas both directly inoculated and contact birds infected with the Δ208–213 mutant shed no detectable infectious virus from the cloaca (Table 2).

**TABLE 2:** Cloacal shedding of wild-type and Δ208–213 viruses from chickens

Virus	Contact or infected chicken	% of birds with virus shedding (no. of birds shedding virus)	Mean duration of shedding (days)	Mean shedding titer (PFU/ml) (±SD)^*a*^
Wild type	Infected	71.4 (5/7)	1.6	8.9 × 10^2^ (±1.6 × 10^3^)
	Contact	28.6 (2/7)	1	5.9 × 10^3^ (±8.0 × 10^3^)
Δ208–213	Infected	0 (0/7)	0	ND
	Contact	0 (0/7)	0	ND

a ND indicates that no shedding was observed, and therefore, the mean titer could not be determined.

Birds were scored daily, in a blind manner, for clinical signs such as respiratory distress; red, crusty, and/or watery eyes; swollen heads; pale wattles; and reduced activity levels. Birds directly inoculated with the wt virus showed some mild clinical signs peaking at days 2 and 3 postinoculation, while birds directly inoculated with the deletion mutant (Δ208–213) virus showed no such signs (Fig. 3D). Contact birds infected with the wt virus exhibited mild to moderate clinical signs peaking at day 2 postexposure, resulting in one bird, which exhibited severe clinical signs, being euthanized for welfare reasons. In contrast, contact birds exposed to the mutant virus showed only mild clinical signs peaking on day 3 postexposure. The most common clinical signs observed in both groups were swollen heads, red eyes, and low activity levels.

Comparative intrahost tropism of the wt and the Δ208–213 deletion mutant virus was evaluated by quantifying the levels of viral M gene RNA in a number of different organs by quantitative reverse transcription-PCR (qRT-PCR); viral M gene RNA levels are a known correlate of infectious virus at early time points (9). On day 2 postinoculation, 3 birds from each group of directly inoculated birds were culled, and tissues were taken. Viral RNA was detected in the nasal tissue and liver in both groups; however, only the wt virus-infected birds had detectable viral RNA in the duodenum, trachea, lungs, and blood (Fig. 3C), and only birds infected with the mutant virus (Δ208–213) had viral RNA in the kidney and spleen. Neither virus was identified in the colon. wt virus RNA was found at higher concentrations in the trachea and lungs than was the Δ208–213 virus (with the mean M gene copy number ranging between 7.7 × 10^5^ and 3.0 × 10^3^ copies of the M gene/μg of total RNA for the wt in the trachea and lungs, compared to <1 × 10^2^ M gene copies/μg of total RNA for the mutant in both organs). RNAs from both viruses were found at comparably high levels in the nasal tissue (3.2 × 10^5^ to 1.9 × 10^5^ copies of the M gene/μg of total RNA for the wt and mutant viruses, respectively).

Although no compensatory changes were seen after passage of the deletion mutants in embryonated eggs, *in vivo* transmission presents much more stringent selective pressure for less fit viruses (39, 40). Therefore, viruses present in buccal swabs from the contact birds of the mutant and wt UDL1/08 groups with the highest levels of shedding were used for sequencing, representing viruses that had undergone two passages in chickens. The HA and NA genes were sequenced to look for compensatory mutations; of the successful reactions (5 out of 7 in the mutant virus group and 7 out of 7 in the wt group), none of the HAs or NAs contained compensatory mutations.

### Mutant viruses show increased avidity for a human-like α2,6-linked sialic acid analogue.

Due to the evidence that residues in the 220 loop can modulate H9 HA receptor binding (18, 29), receptor binding preferences of the wt and deletion mutant viruses were determined by using biolayer interferometry to quantify binding to different avian-like or human-like receptor analogues in a manner similar to that described previously (30).

**FIG 4:**
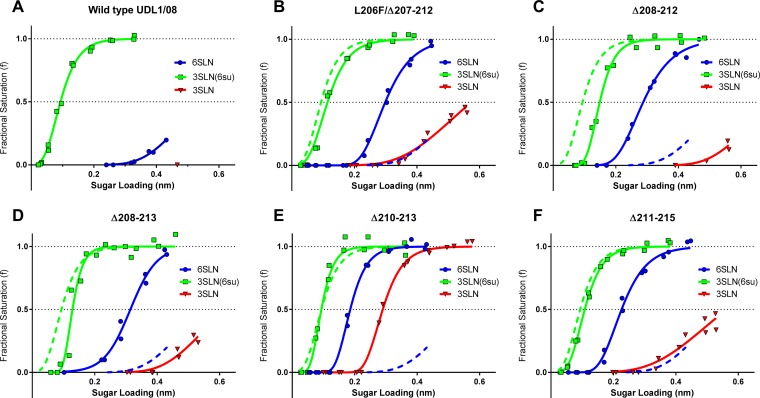
Characterization of the receptor binding properties of 220 loop deletion viruses. Binding of purified virus to three different influenza virus receptor analogues was assayed by biolayer interferometry. All data are modeled with sigmoidal dose-response curves and the amalgamation of two repeats. Dotted curves show binding of the wt virus for direct comparison to the 220 loop deletion viruses. (A) wt UDL1/08 virus; (B) L206F/Δ207–212 mutant; (C) Δ208–212 mutant; (D) Δ208–213 mutant; (E) Δ210–213 mutant; (F) Δ211–215 mutant.

H9N2 viruses of the G1 lineage, such as UDL1/08, were previously shown to have a strong receptor preference for the sulfated variant of the classical avian-like receptor analogue α-2,3-sialyllactosamine (3SLN), here referred to as 3SLN(6su) [Neu5Ac α-2,3 Gal β1,4(6-HSO_3_)GlcNAc]; weak binding to the human-like receptor α2,6-sialyllactosamine (6SLN); and no detectable binding to nonsulfated 3SLN (41). Compared to the wt UDL1/08 virus, all deletion mutants analyzed by using biolayer interferometry showed a substantial increase in binding to the human receptor analogue 6SLN (Fig. 4A to F). The Δ210–213 deletion mutant showed the largest increase in avidity for 6SLN (>75-fold-stronger relative binding), whereas the Δ208–213 mutant showed a modest increase (>7-fold increase) compared to the wt UDL1/08 virus. Additionally, all deletion mutants with the exception of the Δ210–213 mutant showed a significant (>2-fold) decrease in binding to the sulfated avian analogue 3SLN(6su) compared to the wt UDL1/08 virus (a 2.3-fold decrease for the Δ211–215 mutant and a 10-fold decrease for the Δ208–212 mutant). All deletion mutant viruses consistently exhibited some detectable binding to the nonsulfated avian receptor 3SLN, a property that the wt virus completely lacks, suggesting that the deletion mutant viruses generally exhibit less discrimination between different receptor analogues.

### Mutant viruses replicate to higher titers in primary human airway cells.

**FIG 5:**
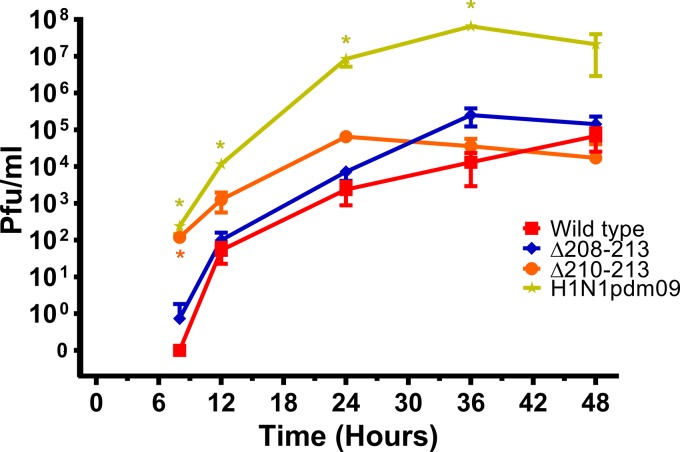
Infection of human primary airway cells with deletion mutant viruses. HAE cultures were infected with 1,000 PFU/transwell. The infectious virus titer was determined by a plaque assay on MDCK cells. Data show means ± standard errors. Significance (* indicates a *P* value of >0.05) was determined by one-way ANOVA with multiple comparisons.

Due to the observed increased binding of the deletion mutant viruses to 6SLN, we were interested to compare the replication kinetics of the wt UDL1/08 virus and the mutant viruses in primary human airway epithelial (HAE) cultures (Fig. 5). We compared the Δ208–213 and Δ210–213 mutant viruses (chosen due to having the most modest and largest increases in 6SLN binding, respectively) to a pandemic H1N1 virus, A/England/195/2009 (here referred to as H1N1pdm09). As expected, H1N1pdm09 replicated to significantly higher titers in human cells than did the avian-origin wt UDL1/08 virus at all time points until 48 h postinfection, with consistent >100-fold-higher titers. At early time points (<36 h postinfection), both mutant viruses grew to titers higher than that of the wt UDL1/08 virus, with titers being between 1.5- and 67-fold higher for the Δ208–213 virus and between 20- and 120-fold higher for the Δ210–213 virus. However, only the Δ210–213 mutant reached a significantly higher titer at any time point (at 8 h postinfection, as determined by one-way ANOVA with multiple comparisons). Overall, these results demonstrated that the deletion mutants had a replicative advantage over the wt UDL1/08 virus in this mammalian *ex vivo* culture system.

### Deletion mutant viruses have lower HA stability than the wild-type virus.

In addition to receptor binding preference, HA pH stability has been repeatedly shown to be an important factor in the adaption of AIVs to efficient airborne transmission in humans or ferrets. AIVs generally have a less stable HA and therefore fuse at a higher pH than human influenza viruses (41, 42). Efficient airborne transmission of AIVs in mammalian model systems (i.e., the ferret) is thought to require mutations in HA that stabilize the molecule to a lower pH of fusion (32, 43). These properties may enable better survival of virions in the harsh microenvironment of respiratory droplets and the acidic mammalian nasal passage.

**FIG 6:**
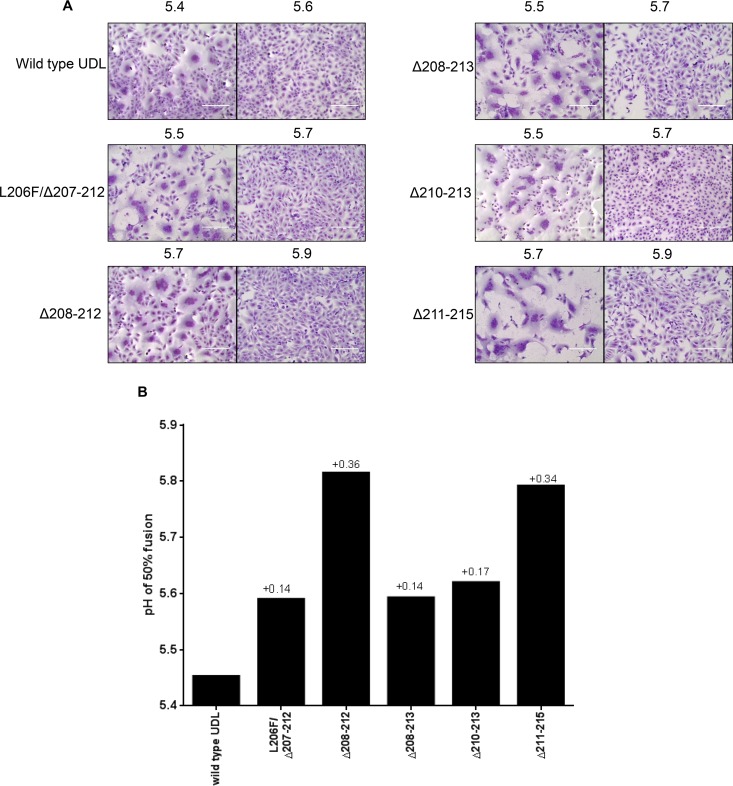
pH stability of 220 loop deletion mutants. (A) Syncytium formation of deletion mutants under different pH conditions (pHs are shown above each image). Shown are data for representative pH conditions where viruses form maximal or minimal syncytia. (B) Summary of predicted pHs of fusion of deletion mutants and the wt virus as determined by 50% syncytium formation in Vero cells. Numbers above the bars show increases in the pH of fusion of mutant viruses compared with the wt virus.

Previous studies have shown that changes at the HA trimer interface, or in the RBS, can modulate pH stability in H5N1 and H7N9 viruses (32, 44, 45). As well as directly measuring pH stability, we also tested the comparative thermal stability, a well-known correlate of pH stability (45, 46), of the mutant viruses. Vero cells were infected with the respective viruses, allowing cell surface expression of HA and NA, followed by treatment with trypsin and then buffers across a pH range to allow syncytium formation. Syncytium formation was quantified as previously described (41). All deletion mutant viruses demonstrated increases in fusion pH (between +0.14 to 0.36 pH units) compared to the wt virus (Fig. 6A and B). Interestingly, there was a good correlation between the size of the deletion and the change in pH stability, with the pairs of viruses bearing deletions of either 5 or 6 amino acids having very similar changes (*P* = 0.0023 by a Student *t* test comparing the effects of the of 5- and 6-amino-acid deletions). Viruses with 6 amino acids deleted had the smallest change in pH stability, while those with 5 amino acids deleted had the largest, and the single 4-amino-acid deletion had intermediate stability. These results indicate that the deletion size, rather than the precise amino acid position, may be the defining factor for the pH stability of these viruses.

**FIG 7:**
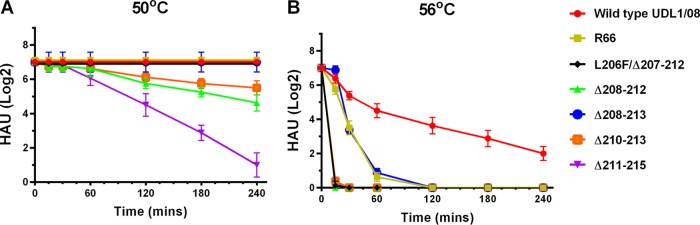
Thermal stability of 220 loop deletion mutants. The thermal stability of hemagglutinin was assayed based on the ability to hemagglutinate chicken red blood cells after incubation at either 50°C (A) or 56°C (B) for a time course.

To test the thermal stability of the deletion mutants, 128 hemagglutination units (HAU) of the wt or deletion mutant viruses were heated at either 50°C or 56°C over a 4-h time course. Samples were taken at regular time points, and a hemagglutination assay was performed to determine changes in virus hemagglutination titers. At 50°C, the Δ211–215, Δ208–212, and Δ210–213 mutants were the least stable viruses, with decreases in hemagglutination titers of between 4- and 64-fold compared to the wt UDL1/08 virus (Fig. 7A). At 56°C, the L206F/Δ207–212 deletion mutant virus also displayed lower stability than the wt virus (Fig. 7B). The Δ208–213 deletion mutant virus, although also less stable than the wt, retained stability comparable to that of Em/R66. As previously reported for multiple influenza virus subtypes (45, 47), we found that thermal stability showed a strong correlation with pH stability (Spearman's rho = 0.92). Overall, these results further demonstrate the destabilizing effect of 220 loop deletions.

## DISCUSSION

Influenza viruses evolve rapidly due to high mutation rates, mutational robustness and plasticity, and the ability to readily reassort (48, 49). Mutations in the HA glycoprotein are known to be particularly influential on modulating the overlapping and interrelated properties of receptor specificity, host and tissue tropism, pathogenicity, and antigenicity (32, 43, 50). Conventional avian influenza vaccines typically lose efficacy as HAs undergo antigenic drift, and several research groups have suggested that the widespread use of poultry vaccines may even contribute to driving antigenic drift (21, 22, 24, 51, 52). Recently, we generated a panel of “antigenic drift-like” escape mutants (through *in vitro* selection with MAbs), some of which contained deletions within the 220 loop region of the HA RBS (34). In this study, we have shown that these loop deletion mutants are still able to replicate to titers comparable to those of wt viruses in chicken cells *in vitro* and *ex vivo*. In an *in vivo* model system, selected deletion mutants were able to replicate and be transmitted between chickens; however, these mutants were slightly attenuated compared to the wt virus. The deletion mutant viruses were able to escape neutralization not only from their cognate selecting MAbs but also from both homologous and heterologous chicken and ferret antisera. In addition, these viruses exhibited various degrees of increased binding to the human-like α2,6 receptor analogue and greater replicative ability in primary human airway cells than the wt virus, indicating possibly increased zoonotic potential. However, the deletion mutants generally had lower pH and thermal stability than the wt virus, a property that may increase their pathogenicity in poultry (53, 54) but would likely limit their pandemic potential without compensatory stabilizing mutations (32, 55).

Our results suggest that HA deletion mutant viruses such as these may be slightly attenuated; they could potentially outcompete wild-type viruses in vaccinated poultry while incidentally modulating other viral phenotypes such as receptor binding and pH stability. The possibility that vaccine-induced immunity may influence antigenic drift was previously explored through studies with comparative molecular epidemiology; however, the underlying process remains poorly understood and merits further investigation (22, 51, 56). Both deletion mutant viruses tested here for their growth in primary human airway cells, Δ208–213 and Δ210–213, exhibited increased replication compared to the wt UDL1/08 virus. The Δ208–213 mutant showed only a mild increase in replication compared to the wt UDL1/08 virus and only a moderate increase (>7-fold) in 6SLN binding. The Δ210–213 mutant showed a larger increase in replication and as well as a larger increase in 6SLN binding (>75-fold). As expected, this demonstrates that 6SLN binding correlates well with replication in primary human cells. Neither deletion mutant, however, grew to levels comparable to those of human H1N1pdm09. There could be multiple reasons for this; for example, although both deletion mutants displayed some avidity toward 6SLN, this level of binding was still relatively low compared to that of human H1N1pdm09 viruses (41). Additionally, UDL1/08, being an avian-adapted virus, does not naturally possess many of the molecular markers associated with mammalian adaption in H1N1pdm09 and other human influenza viruses, for example, PB2-Q591K, PB2-E627K, or PB2-D701N (57, 58).

**FIG 8:**
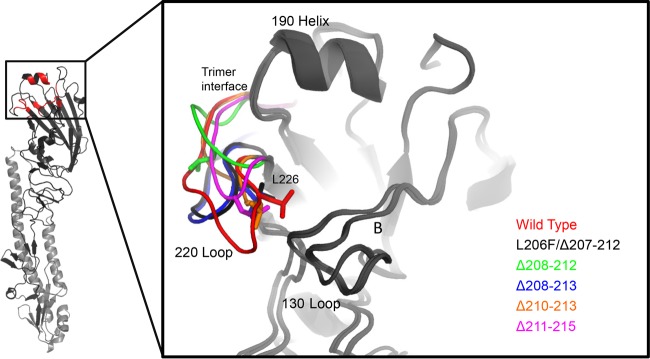
Modeling of 220 loop deletion mutants. Shown are aligned predicted structures of HA receptor binding sites in wt UDL1/08 and 220 loop deletion mutants. Structural predictions were made by using Phyre2 (73) (Imperial College London and BBSRC). Structural alignments were made by using PyMOL (Schrödinger LLC) (74).

To explain how changes in the phenotypes of deletion mutants may be linked to changes in structure, we utilized homology modeling conducted with the Phyre2 protein fold recognition server. Initially, we threaded the sequence of UDL1/08 HA (minus the transmembrane domain and cytoplasmic tail) to the only currently solved H9 HA structure (a BJ94-lineage virus, A/Swine/Hong Kong/9/98) (PDB accession number 1JSD). We then aligned the predicted structures of the deletion mutants and compared the projected conformations of the 220 loop regions. Notably, the 220 loop region, which forms a key region of the antigenic site H9-A (34), is anticipated to be in a different conformation in all deletion mutant structures compared to the wt UDL1/08 virus. This property may explain the observed changes in the antigenicity of the deletion mutant viruses (Fig. 8). Additionally, several viruses were predicted to have structural variations at the trimer interface that may modulate pH stability (59, 60). All predicted structures showed a different conformation of residues 216 and 217 (residues 226 and 227 according to H3 numbering), both of which are known to modulate H9 HA receptor binding specificity (18, 29).

One interesting feature of the Δ208–213 deletion mutant virus, when tested *in vivo*, was its complete lack of cloacal shedding and intestinal tropism. We speculate that the deletion mutant failed to disseminate into the gastrointestinal tract due to insufficient replicative fitness, as evidenced by the low virus titers observed at early time points from buccal swabs. Another possibility is that the deletion mutant's lower pH stability, shifted receptor binding profile (away from sulfated 3SLN and toward 6SLN), or a combination of these factors did not favor transport through the highly acidic gizzard and subsequent attachment to and replication in the gut epithelium (known to contain high α2,3- and low α2,6-SA levels) (61, 62). The apparent lack of intestinal tropism found in the mutant virus, combined with the 100% transmission rate, again shows the apparent dispensability of transmission through the oral-fecal route in our model system (9).

Naturally occurring H9N2 virus isolates containing 220 loop deletions have been reported from China (6) and, very recently, from Pakistan (63). The report from China described a BJ94-lineage virus, A/chicken/Shandong/818/2012 (SD/818), with a single amino acid deletion at position 217 (H9 mature numbering; position 227 according to H3 numbering), that was isolated from commercial chicken flocks showing high morbidity and mortality rates. The SD/818 virus was subsequently evaluated to be moderately pathogenic under laboratory conditions in SPF birds with an intravenous pathogenicity index (IVPI) score of 1.0 (high pathogenicity is defined as an IVPI score of >1.2). In comparison, a genetically closely related H9N2 virus with a full-length HA not harboring the 220 loop deletion had an IVPI score of 0 (7, 58). We speculate that the increased pathogenicity of SD/818 may be due to a higher pH of fusion of its HA molecule, since previous studies have shown that an unstable HA molecule with a high pH of fusion correlates well with virus pathogenicity in avian hosts (53, 54). Our present study suggests that deletions in the 220 loop may incur a stability cost; however, data on the HA stability phenotype of SD/818 are lacking, nor is there information regarding the vaccination status of the birds from which this virus was isolated. Furthermore, there has been a preliminary report of a mutant H9N2 virus, from Pakistan, that has 6 amino acids deleted in the 220 loop region (63), further supporting the hypothesis that these viruses may arise spontaneously in poultry.

Other virus subtypes with 220 loop deletions (notably H3 and H7) have been generated *in vitro* as well as being found in the field. During an escape mutant analysis similar to our original study (34), Daniels et al. generated a mutant of H3N2 laboratory strain X-31 with a 7-amino-acid deletion from positions 214 to 220 (equivalent H9 mature numbering; positions 224 in 230 according to H3 numbering) (64). This H3 deletion mutant exhibited altered receptor binding compared to wild-type strain X-31, such that instead of a typical α2,6 binding preference, the mutant bound both α2,6- and α2,3-linked SA, thereby losing its preference toward one sugar or the other, in a manner similar to that of our H9N2 deletion mutants. Additionally, the X-31 deletion mutant showed an increase in the pH of fusion of 0.2 pH units, indicating reduced HA stability, again similar to our H9 220 loop deletion mutants.

Between 1994 and 2006, a sublineage of low-pathogenicity H7N2 viruses that harbored an 8-amino-acid deletion in the 220 loop (positions 211 to 218, equivalent to H9 mature numbering; positions 221 to 228 according to H3 numbering) circulated in chickens on the East Coast of the United States. These H7 viruses were shown to have a higher propensity for binding human-like receptors by glycan microarray analysis than other contemporary avian H7 viruses and were able to efficiently replicate in ferrets (37, 65, 66). A single human case of infection by this H7 virus was reported previously (67). A structural study by Yang et al. suggests that HA was able to functionally compensate for the loss of a 220 loop through substitutions in peripheral residues around the RBS (37), a feature that we did not observe for our mutants after either *in ovo* or *in vivo* passage.

To conclude, our findings highlight the importance of continued vigilant surveillance of avian populations for the emergence of novel influenza virus variants, particularly in vaccinated populations. If viruses are identified with deletions in the 220 loop region (particularly in H5, H7, or H9 viruses), these isolates should be further evaluated for their zoonotic risk potential through assessments of receptor binding preference and increased replication in mammalian models. The emergence of viruses with 220 loop deletions in poultry should also trigger a reassessment of vaccine efficacy, as our data suggest that loop deletion viruses are likely to exhibit markedly different antigenicities compared to those of closely related progenitor viruses. Without further stabilizing mutations in HA, we suggest that 220 loop deletion viruses, although they may pose an increased zoonotic risk compared to other field strains, are unlikely to be able to be transmitted from human to human by the airborne route and therefore are unlikely to pose a particularly elevated pandemic threat. Overall, this study contributes to the body of literature on AIV molecular determinants associated with zoonotic risk and emergence and underscores the complexity of predicting which field strains are most likely to predominate or may develop the capacity for human-to-human transmission.

## MATERIALS AND METHODS

### Ethics statement.

All animal studies and procedures were carried out in strict accordance with European and United Kingdom Home Office regulations and the Animals (Scientific Procedures) Act 1986 Amendment Regulations 2012. This work was carried out under UK Home Office-approved project license numbers 30/2683 and 30/2952. As part of this process, the work was subjected to scrutiny and approval by the Ethics Committee at The Pirbright Institute.

### Cells, eggs, and tissues.

MDCK cells, HEK 293T cells, and Vero cells were maintained in Dulbecco's modified Eagle medium (DMEM) supplemented with 10% fetal bovine serum (FBS) at 37°C in 5% CO_2_. Virus was grown in 10-day-old SPF embryonated eggs and harvested at 48 h postinoculation. CKCs and eTOCs were prepared as previously described (68, 69). Primary human airway epithelial cultures, MucilAir, were purchased from Epithelix SAS (France). Cells were obtained from donor pools of nasal tissue and were maintained in MucilAir culture medium at 37°C in 5% CO_2_.

### Recombinant viruses and virus rescue.

H9N2 avian influenza virus UDL1/08 was used throughout this study unless otherwise stated. UDL1/08 was rescued by using a standard influenza virus 8-plasmid bidirectional reverse-genetics system as previously described (70). Loop deletion HA plasmids of UDL1/08 and the other H9N2 viruses of BJ94 (A/chicken/Wenzhou/606/2013) and American (A/turkey/Wisconsin/1/1966) lineages were generated by site-directed mutagenesis as described previously (34). High-egg-growth reassortant viruses were generated by rescuing HA and NA of H9N2 viruses with the remaining 6 genes of the high-egg-growth strain A/Puerto Rico/8/1934 H1N1 (PR8), here referred to as 2:6 reassortants. Non-UDL1/08 H9N2 viruses were rescued from their HA rescue plasmid with the NA plasmid from UDL1/08 and the remaining genes from PR8. Viruses were titrated by a virus plaque assay or a 50% tissue culture infective dose (TCID_50_) assay (in the case of microneutralization assays) as previously described (71). Deletion mutant PFU and TCID_50_ values were relative for all viruses tested.

### Hemagglutination inhibition and microneutralization assays.

Hemagglutination inhibition assays and microneutralization assays were carried out by using 2:6 reassortants and whole UDL1/08 virus, respectively. Both assays were performed as previously described (71). The HI assay utilized 1% chicken red blood cells.

### Virus growth curves.

MDCK cells and primary chicken kidney cells were infected with recombinant whole UDL1/08 wt and mutant viruses at MOIs of 0.001 and 0.01, respectively (240 or 2,400 PFU/6-well plate well) for 1 h at 37°C. Cells were then washed twice with phosphate-buffered saline (PBS) to remove unbound virus, and 2 ml of virus growth medium (DMEM plus 2 μg/ml tosyl phenylalanyl chloromethyl ketone [TPCK]-treated trypsin for MDCK cells and Eagle's minimum essential medium [EMEM], 7% bovine serum albumin [BSA], and 10% tryptose phosphate broth [TPB] for CKCs) was added. Infected culture medium was harvested at 12, 24, 48, and 72 h postinfection, and virus titers were estimated in triplicate by using MDCK cell-based plaque assays. eTOCs were infected in sextuplicate with 240 PFU of virus/eTOC and sampled at 12, 18, 24, and 36 h postinfection. For growth curves in human airway epithelial (HAE) cultures, cells were first washed with serum-free medium to remove excess mucus and then infected with 1,000 PFU/transwell at the apical surface. Virus was collected by washing the cells at the apical surface with 200 μl of serum-free medium at 8, 12, 24, 36, and 48 h postinfection.

### Passage of virus in eggs.

Whole virus was diluted in a 10-fold serial dilution in PBS; each dilution was added to a single egg. Eggs were harvested at 2 days postinoculation and tested for the presence of virus by hemagglutination. For each virus, the highest dilution with a positive HA titer was then used for further passage and/or sequencing.

### Sequencing of virus.

Sequencing of virus HA and NA genes was performed as described previously (34). Briefly, RNA from viruses was extracted from swab-soaked viral transport medium, egg allantoic fluid, or cell culture medium by using the QIAamp viral RNA minikit (Qiagen). A Verso cDNA synthesis kit (Thermo Scientific) was used to perform reverse transcription with the universal FluA primer. PCR was undertaken by using primers specific for HA1, HA2, and NA adjuncted with 3′ M13F and M13R sequencing motifs (sequences of primers are available upon request). The QIAquick PCR purification kit (Qiagen) was used to purify the resulting products, which were then sequenced by using the M13F and M13R primers.

### Animal studies.

For *in vivo* studies on the comparative properties of the Δ208–213 loop deletion virus and the wt UDL1/08 virus, 3-week-old specific-pathogen-free Rhode Island Red birds were housed in isolators, with 7 birds per isolator, 1 day prior to infection. At day 0, birds were infected intranasally with 10^6^ PFU of virus per bird. On day 1 postinfection, seven contact birds were added to each isolator. Cloacal and oropharyngeal swabs were obtained from all birds on days 0 to 6, 8, and 10, and swabs were soaked and vortexed in virus transport medium (medium 199, 0.5% BSA, 2 × 10^6^ U penicillin G [Sigma], 200 mg/liter streptomycin [Sigma], 2 × 10^6^ U/liter polymyxin B [Sigma], 250 mg/liter gentamicin [Sigma], 5 × 10^5^ U/liter nystatin [Sigma], 60 mg/liter ofloxacin HCl [Sigma], and 200 mg/liter sulfamethoxazole [Sigma]). The viral titer of the inoculum in PFU was then determined on MDCK cells. On day 2, three directly infected birds from each group were culled by pentobarbital overdose, and tissues were removed and frozen in RNAlater (Life Technologies). At 14 days postinfection, all birds were euthanized by pentobarbital overdose and were bled for serum preparation.

### Detection of virus in animal tissues.

Tissues from infected and control birds were homogenized in TRIzol (Life Technologies). The aqueous fraction was then extracted after mixing with chloroform and centrifugation. An RNeasy minikit (Qiagen) was then used for subsequent steps of RNA extraction. qRT-PCR was performed by using a Superscript III Platinum One-Step qRT-PCR kit (Life Technologies). Primer and probe sets against the M gene were used as previously described (9, 72). Reaction conditions used were 50°C for 5 min and 95°C for 2 min followed by 40 cycles of 95°C for 3 s and then 60°C for 30 s. T7-transcribed RNA standards of the M gene were used to generate a standard curve to quantify observed M gene copy numbers.

### Homology modeling of hemagglutinin structures.

Structural prediction of H9 HA and derivative loop deletion mutants of UDL1/08 was performed by using Phyre2 (http://www.sbg.bio.ic.ac.uk/phyre2) (73). Images were generated and rendered in PyMOL (https://www.pymol.org/) (74).

### Thermal stability assay.

2:6 recombinant H9N2 viruses were diluted to a stock of 128 HAU/50 μl. Virus was then heat treated on a heat block at either 50°C or 56°C for various times (0, 15, 30, 60, 120, 180, and 240 min). Heat-treated viruses were then assayed for HA titers in quadruplicate.

### Syncytium formation assays.

Syncytium formation assays were performed as previously described (41). Vero cells were virally infected with a high MOI of >3. At 16 h postinfection, cells were treated with trypsin for 15 min and then exposed to morpholineethanesulfonic acid (MES) buffers adjusted across a pH range. At 3 h posttreatment, cells were fixed and stained with Giemsa stain (Sigma-Aldrich). Images were taken on the Evos XL cell imaging system (Life Technologies). Percent syncytium formation was calculated from 5 random fields per virus per well. The predicted pH of fusion was calculated from fitting sigmoidal dose-response curves to the data by using GraphPad Prism version 6 (GraphPad Software).

### Virus purification.

Virus from clarified egg allantoic fluid was initially pelleted by ultracentrifugation at 27,000 rpm for 2 h. Pellets were resuspended in PBS and homogenized by using a glass homogenizer. The virus suspension was then centrifuged on a continuous 30 to 60% sucrose gradient at 27,000 rpm for 2 h. The virus-containing band was then diluted in PBS, and virus was pelleted once again at 27,000 rpm for 2 h. Finally, virus was resuspended in PBS–0.01% azide. The virus concentration was determined by using an enzyme-linked immunosorbent assay (ELISA) against the virus nucleoprotein (NP) as described previously (30).

### Biolayer interferometry.

Binding of virus to sialic acid receptor analogues was measured by using an Octet Red biolayer interferometer (Pall FortéBio) as previously described (41). Briefly, receptor analogues of sialoglycopolymers consisting of a 30-kDa polyacrylamide backbone conjugated to 20 mol% trisaccharide 6SLN, 3SLN, or 3SLN(6su) and 5 mol% biotin (Lectinity Holdings). Sialylglycopolymers were immobilized on streptavidin-coated biosensors (Pall FortéBio) at concentrations ranging from 0.01 to 0.5 μg/ml in a solution containing 10 mM HEPES (pH 7.4), 150 mM NaCl, 3 mM EDTA, and 0.005% Tween 20 (HBS-EP). Virus was diluted in HBS-EP containing 10 μM oseltamivir carboxylate (Roche) and 10 μM zanamivir (GSK) to a concentration of 100 pM. The association of virus with the immobilized receptors was measured at 20°C for 30 min. Virus binding amplitudes were normalized to fractional saturation and plotted as a function of sugar loading. Virus relative estimated dissociation constants (*K_D_*s) were calculated as described in our previous study (41).
